# Maternal awareness of young children’s physical activity: levels and cross-sectional correlates of overestimation

**DOI:** 10.1186/1471-2458-13-924

**Published:** 2013-10-03

**Authors:** Kathryn R Hesketh, Alison M McMinn, Simon J Griffin, Nicholas C Harvey, Keith M Godfrey, Hazel M Inskip, Cyrus Cooper, Esther MF van Sluijs

**Affiliations:** 1 UKCRC Centre for Diet and Activity Research, Institute of Public Health, University of Cambridge, Cambridge, UK; 2 MRC Epidemiology Unit, University of Cambridge, Cambridge, UK; 3 MRC Lifecourse Epidemiology Unit, University of Southampton, Southampton General Hospital, Southampton, UK; 4 Southampton NIHR Biomedical Research Unit in Nutrition, Diet and Lifestyle, Southampton General Hospital, Southampton, UK

**Keywords:** Physical activity, Awareness, Preschool children

## Abstract

**Background:**

Factors associated with parental awareness of children’s physical activity (PA) levels have not been explored in preschool-aged children. This paper investigates maternal awareness of preschool-aged children’s PA levels and determined correlates associated with maternal overestimation of PA.

**Methods:**

Data from the Southampton Women’s Survey, a UK population-based study, were collected March 2006 through June 2009. Daily minutes of moderate-to-vigorous PA (MVPA) were derived using accelerometry in 478 4-year-old children. Mothers who were realistic or overestimated their child’s PA were identified. Log-binomial regression was used to analyse correlates of maternal overestimation of PA levels in children whose mothers perceived them to be active (n = 438).

**Results:**

40.8% of children were classified as inactive: 89.7% of these were perceived to be active by their mothers (over-estimators). These mothers were more likely to think their child sometimes lacked skills required to be physically active (RR (95% CI) = 1.29(1.03-1.63)) and their child was more likely to attend nursery full-time (RR = 1.53(1.14-2.04)). They were less likely to have older children at home (RR = 0.71(0.56-0.90)).

**Conclusions:**

Almost 90% of mothers of inactive preschool-aged children perceive their child to be active. Nursery-school attendance and having older siblings at home may be important to consider when designing behavioural interventions to increase PA in preschool children.

## Background

Physical activity plays an important role in the maintenance of health, with active children tending to have decreased adiposity [1,2], fewer cardiovascular disease risk factors [3] and improved bone health [4] compared to their less active peers. Physical activity in preschool children may be particularly important as it has been associated with improved gross motor control [5] and aids the development of basic movement patterns that form the foundation of a wide range of movements later in life [6].

Levels of physical activity are known to decrease from late childhood into adolescence [7,8]. Recently developed recommendations for preschoolers in a number of countries advocate 180 minutes of activity per day at any intensity [9-11], with previous guidelines suggesting engagement in 60 minutes of moderate-to-vigorous physical activity (MVPA) each day [6]. Studies have provided wide-ranging estimates as to how active three- to five-year-old children are: most suggest children engage in little MVPA [12-14] and that the new guidelines of 180 minutes may also to be difficult for children to meet [13]. As more favourable health outcomes have been related to physical activity of higher intensity in preschool aged children [15,16] the importance of activity intensity for the youngest age groups is still under debate.

Interventions to increase levels of physical activity in young children may be beneficial, and although a growing number have been developed to increase physical activity in preschool children, outcomes have been mixed [17,18]. The underlying reasons for variable intervention effects remain unclear, but both children’s and parents’ (lack of) awareness of physical activity levels may be an important determinant. Awareness of behaviour may be particularly relevant for complex, often habitual, health behaviours like physical activity [19]. Should people be unclear where the threshold lies between healthy and unhealthy behaviours [20], they may deem their own, or their child’s behaviour, to be healthier than it is. For example, adults who tend to overestimate their personal activity levels have been shown to have a lower BMI and better perceived health [20-22] compared to those who are realistic about their low levels of activity. These adults are unlikely to perceive a need for behaviour change and may therefore be less inclined to increase their low physical activity levels.

Parents have considerable influence over their children’s behaviours. The effect of factors such as positive role modelling, providing instrumental and social support, and parental participation on children’s activity levels have been frequently studied in preschool-aged children [23,24]. Several studies conducted in school-aged children suggest nearly 80% of parents of inactive children perceived their child to be sufficiently active [25,26], and that lack of awareness of inactivity is higher in parents than it is in children [25]. This may prevent parents engaging in interventions to modify their children’s behaviour [27], with few intervention studies currently taking parental awareness into account [28]. Further, parental awareness of their child’s behaviour may be particularly important in preschool-aged children given their young age and own lack of self-awareness [29].

This study aims to assess maternal awareness of physical activity levels in a population-based sample of four-year-old British children and determine biological, behavioural and psychological correlates of maternal overestimation. This will help establish whether similar associations as previously reported for older children are observed and identify correlates potential targets for future intervention.

## Methods

The Southampton Women’s Survey is a population-based prospective cohort study based in Southampton, UK. Details of the cohort have been published previously [30]. The study was designed to assess maternal diet and lifestyle before and during pregnancy, with any subsequent live births followed to examine how children’s pre-natal development interacts with their postnatal growth, and how both may affect their risk factors for a range of future chronic diseases [30]. Parents of all participating children gave full and informed written consent. The Southampton and South West Hampshire Local Research Ethics Committee granted ethical approval for the study.

At age four, a subsample of 730 children participated in a secondary study to investigate potential correlates of physical activity. At this four-year-old visit, conducted between March 2006 and June 2009, objective measurement of physical activity was undertaken (n = 594) and a questionnaire was completed by the child’s mother (n = 569). This questionnaire has been validated in a subset of the cohort [31] with the specific measures used in analyses described below.

### Physical activity

Physical activity was measured using the Actiheart, a lightweight combined heart rate monitor and accelerometer (Cambridge Neurotechnology Ltd, Papworth, UK), validated in preschool children [32,33]. The Actiheart was attached to the chest and participants were instructed to wear the monitor continuously for seven days, including when sleeping and during any water-based activities. Due to storage capacity, the monitor was set to record at 60-second epochs to allow for 7-day assessment. Only accelerometry data were used for this analysis, as is appropriate in this age-group [33,34]. Data were analysed using a program (MAHUffe: [35]; which enabled removal of any data recorded between 10 pm and 6 am (defined as sleep based on the investigation of hourly activity levels), periods of 100 minutes with continuous zero-activity counts [36], and days with <600 minutes of recording (the cut-off to define a valid day). Participants with ≥3 days of valid physical activity data, including at least one weekday and one weekend day, were included. Average daily minutes of moderate to vigorous physical activity (MVPA) were calculated for each child, with MVPA defined using a cut-off ≥400 counts per minute. This 400-count Actiheart cut-point, derived and validated experimentally in children and adolescents [37,38], roughly equates to a cut-point of 2000 counts in the Actigraph 7164 accelerometer (Actigraph, Pensacola, FL, USA).

### Derivation of awareness categories

Four awareness categories were derived using child’s objective physical activity level, and maternal perception of that activity (Table 1). Children were classified as active or inactive based on whether or not they engaged in an average of 60 minutes of MVPA per day. These were the activity recommendations for preschool-aged children at the time of measurement, [6,39] and therefore the guidelines parents were exposed to. Sensitivity analyses were also conducted to analyse the number of children categorised as 'active’ using a cut-off of ≥55 minutes or ≥65 minutes compared to ≥60 minutes used here. Maternal perception of their child’s physical activity level was assessed using the question: “Would you describe your child as physically active?” Response categories ranged on a five-point scale from strongly agree to strongly disagree. Those who agreed with the statement were combined, as were those who did not or had no strong opinion (“neither agree nor disagree”), to give a response of perceived active, or inactive, respectively [25].

**Table 1:** Derivation of maternal awareness category by comparing maternal rated and accelerometer assessed physical activity levels (n = 478)

**n (%)**	**Maternal rated PA levels**		
	**Perceived inactive**^**a**^	**Perceived active**^**b**^	**Total**
Inactive^c^	20 (4.2)	175 (36.6)	195 (40.8)
	*Realistic Awareness (Inactive)*	*Unaware (Overestimation)*	
Active^d^	20 (4.2)	263 (55.0)	283 (59.2)
	*Unaware (Underestimation)*	*Realistic Awareness (active)*	
Total	40 (8.4)	438 (91.6)	478 (100)

^a^Mother strongly/disagreed or had no view with statement “Would you describe your child as physically active?”; ^b^Mother strongly/agreed with statement “Would you describe your child as physically active?”; ^c^<60 minutes of accelerometer derived MVPA per day; ^d^≥60 minutes of accelerometer derived MVPA per day; PA: physical activity; MVPA: Moderate and vigorous PA.

### Exposure variables

Data on potential correlates, which were chosen as plausible predictors of overestimation, were obtained from the questionnaire. Ethnicity was reported (96.6% white) but then excluded from analyses due to homogeneity, as was home ownership (87.3% owned their home). Self-reported age at which mothers left full-time education was categorized into: ≤16 years, 17–18 years and >18 years. Maternal activity was assessed using a validated questionnaire [40]: information about occupational activity level, and weekly hours spent cycling and performing other exercise over the previous 12 months was used to assign mothers to one of four categories: inactive, moderately inactive, moderately active, and active.

Day care, nursery or preschool attendance was classified as either *full-time* (≥30 hours per week) or *part-time or other* (<30 hours per week). Two dichotomous variables were also created for whether a child had older (yes/no) and younger (yes/no) siblings living at home. Maternal perception of their child being well-behaved, their child’s enjoyment of physical activity and perceived importance of physical activity, and whether their child was outgoing or restless were all measured on a five point Likert scale ranging from *strongly disagree* to *strongly agree*. Two further questions assessed the frequency with which mothers perceived their child’s physical activity to be limited due to child’s disinterest or a lack of skill on a 5-point response scale (*never* to *very often)*. These categorical variables were dichotomised for analysis.

Child’s age and gender, along with height assessed using a Leicester height measure and weight assessed using calibrated digital scales (Seca, Ltd., Birmingham, UK) [41] were recorded during the visit. Mother’s height and weight were also measured at this time. Height and weight were then used to calculate child’s and mother’s BMI (kg/m^2^) and subsequently child’s BMI z-score [42].

### Statistical analysis

Data were analysed in 2011 using Stata/SE 11. Characteristics of those included in and excluded from analyses and characteristics of boys and girls were compared using *t-*tests or chi-square tests. Due to the low proportion of mothers who perceived their child to be inactive (n = 40), there was insufficient variability in the data to conduct analyses with four separate categories. Only those children whose mothers perceived them to be active (realistic active (n = 263) and over-estimators (n = 175)) included in the regression models. Analyses therefore explored associations with inactivity among children whose mothers perceived them to be active (referred to as overestimation for the purpose of this paper). Unadjusted associations between potential biological, social and behavioural correlates and overestimation of child’s physical activity were assessed using log-binomial regression. This is a generalized linear model, which uses a logarithmic link function and binomial distribution for the residual [43-45]. This method was chosen because the odds ratios obtained from logistic regression can often overestimate the prevalence ratio in cross-sectional studies [46]. Further, it has been suggested that controlling for confounders of the odds ratio (using logistic regression) is not the same as doing so for the prevalence ratio [47,48]. Therefore, log-binomial regression presents a conservative, more interpretable prevalence ratio output compared to the odds ratio of logistic regression. All variables that were significantly associated with overestimation in the unadjusted models were carried forward and entered simultaneously into a multiple regression analysis. Variables were then removed from the adjusted model if they did not meet the pre-defined significance level of p < 0.05.

## Results

The final sample, with both questionnaire and valid physical activity (≥3 days) data, consisted of 478 participants. No significant differences in child’s age or sex, or maternal age, BMI and age leaving education were evident between those included in and excluded from analyses (n = 83). Descriptive characteristics of the sample by physical activity awareness category are presented in Table 2.

**Table 2:** Participant and descriptive data classified by maternal awareness of child’s physical activity level (n = 478)

	**Realistic inactive**	**Under-estimators**	**Realistic active**	**Over-estimators**
*Child characteristics*				
Number of participants (Female)	20 (16)	20 (10)	263 (129)	175 (97)
Age (years)	4.1 ± 0.1	4.1 ± 0.1	4.1 ± 0.1	4.1 ± 0.1
BMI (kgm^-2^)	16.6 ± 1.8	15.9 ± 2.0	15.9 ± 1.3	16.0 ± 1.3
BMI z-score	0.56 ± 1.1	0.02 ± 1.3	0.08 ± 0.93	0.14 ± 0.98
Has an older sibling living at home	9 (45)	9 (45)	137 (52)	69 (39)
Has a younger sibling living at home	10 (50)	13 (65)	109 (41)	79 (45)
Attends nursery				
Full-time	2 (10)	0 (0)	14 (5)	20 (11)
Part-time	18 (90)	20 (100)	246 (95)	152 (89)
*Maternal characteristics*				
Age (years)	34.8 ± 3.1	34.3 ± 3.5	35.3 ± 3.6	35.2 ± 2.7
BMI (kgm^-2^)	26.3 ± 5.8	27.7 ± 7.5	26.5 ± 5.2	26.5 ± 5.7
Age leaving full-time education (years)				
≥16 years	3 (15)	8 (40)	86 (33)	52 (30)
17-18 years	5 (25)	6 (30)	87 (33)	71 (41)
>18 years	12 (60)	6 (30)	84 (32)	51 (29)
Physical activity level				
Inactive	3 (15)	4 (20)	43 (16)	31 (18)
Moderately inactive	8 (40)	10 (50)	106 (40)	69 (39)
Moderately active	7 (35)	2 (10)	52 (20)	44 (25)
Active	2 (10)	4 (20)	56 (21)	25 (14)
*Maternal perception*†				
Child is outgoing				
Strongly/disagree/neither	10 (50)	11 (55)	63 (25)	46 (27)
Strongly/agree	10 (50)	9 (45)	191 (75)	124 (73)
Child is restless				
Strongly/disagree/neither	14 (70)	9 (45)	138 (54)	100 (59)
Strongly/agree	6 (30)	11 (55)	117 (46)	70 (41)
Child is well behaved				
Strongly/disagree/neither	4 (20)	6 (30)	69 (27)	30 (18)
Strongly/agree	16 (80)	14 (70)	185 (73)	140 (82)
Child enjoys PA				
Disagree/neither	17 (85)	16 (80)	85 (32)	62 (35)
Strongly/agree	3 (15)	4 (20)	178 (68)	113 (65)
PA is important				
Disagree/neither	9 (45)	12 (60)	78 (30)	49 (28)
Strongly/agree	11 (55)	8 (40)	184 (70)	124 (72)
Physical activity is limited because:				
Child is not interested in physical activity				
Never	3 (15)	4 (20)	125 (49)	85 (49)
Rarely/sometimes/often/very often	17 (85)	16 (80)	129 (51)	90 (51)
Child doesn’t have the skills				
Never	10 (50)	10 (50)	186 (73)	110 (63)
Rarely/sometimes/often/very often	10 (50)	10 (50)	69 (27)	65 (37)

Values are n (%) or mean ± SD unless stated otherwise; † Variables dichotomised; SD, standard deviation; BMI, Body Mass Index; PA, Physical Activity.

Fifty-nine per cent of parents accurately reported their child’s level of physical activity (55.0% realistically active; 4.2% realistically inactive). Of the 40.8% of inactive children, 89.7% of parents (36.6% of all parents) thought that their children were active. A number of parents with active children (7.1%; 4.2% of all parents) deemed their child to be inactive. Interestingly, regardless of children’s actual activity levels, parents who perceived their children to be inactive were less likely to report that their child was outgoing or enjoyed physical activity, or that physical activity was important. They were also more likely to report children showed a lack of interest in physical activity or lacked skills to be physically active.

Of the children whose mothers perceived them to be active (n = 438), 40% were classified as an inactive using an objective measure of physical activity (over-estimators). Unadjusted and adjusted associations for each of the exposure variables with maternal overestimation are shown in Table 3. Of the 16 exposure variables, four were significantly associated with overestimation in the unadjusted analyses, three of which remained statistically significant in the multiple regression analysis. In the final model, having no older siblings, full-time nursery attendance and maternal perception that their child on occasion lacked skills to be physically active were all significantly associated with maternal overestimation of their child’s physical activity. Sensitivity analyses showed that although using a MVPA threshold of >55 or >65 minutes changed the prevalence of overestimation (to 34% and 46% respectively), this did not impact conclusions drawn from the regression analyses.

**Table 3:** Associations between child and maternal characteristics and maternal overestimation of their child's physical activity level (n = 438)

**Variable**	**Unadjusted RR**	**Adjusted RR**
	**(95% CI)**	**(95% CI)**
*Child characteristics*		
Sex (ref: Male)	1.17 (0.93, 1.47)	
Age (in years)	0.74 (0.12, 4.64)	
BMI z-score	1.05 (0.93, 1.18)	
Has an older sibling living at home (ref: no older sibling)	0.73 (0.57, 0.93)*	0.71 (0.56, 0.90)**
Has a younger sibling living at home (ref: no younger sibling)	1.08 (0.86, 1.37)	
Attends nursery (ref: part-time)		
Full-time	1.54 (1.13, 2.10)*	1.53 (1.14, 2.04)**
*Maternal characteristics*		
Age (in years)	1.00 (0.97, 1.03)	
BMI (kgm^-2^)	1.00 (0.98, 1.02)	
Age leaving full-time education (ref: 16 or younger)		
17 or 18 years	1.19 (0.91, 1.57)	
19 years or older	1.00 (0.74, 1.36)	
Physical activity level (ref: Inactive)		
Moderately inactive	0.94 (0.68, 1.30)	
Moderately active	1.09 (0.77, 1.55)	
Active	0.74 (0.48, 1.12)	
*Maternal perception*		
Child is outgoing (ref: Strongly/disagree/neither)		
Strongly/agree	0.93 (0.72, 1.21)	
Child is restless (ref: Strongly disagree/disagree/neither)		
Strongly/agree	0.85 (0.68, 1.09)	
Child is well behaved (ref: disagree/neither)		
Strongly/agree	1.42 (1.03, 1.97)*	
Child enjoys PA (ref: Disagree/neither)		
Strongly/agree	0.92 (0.73, 1.17)	
PA is important (ref: Disagree/neither)		
Strongly/agree	1.04 (0.81, 1.35)	
Physical activity is limited because:		
Child is not interested in physical activity (ref: never)		
Rarely/sometimes/often/very often	1.02 (0.81, 1.28)	
Child doesn’t have the skills (ref: never)		
Rarely/sometimes/often/very often	1.31 (1.04, 1.64)*	1.29 (1.03, 1.63)*

RR, rate ratio; CI, confidence interval; ref, reference category; BMI, Body Mass Index; PA, Physical Activity. Model 2: Adjusted for nursery attendance, older sibling at home and maternal perception of physical activity skills. *p ≤ 0.05; **p < 0.005.

## Discussion

This study assessed maternal awareness of preschool children’s physical activity and investigated correlates of overestimation in a population-based sample of four-year-old British children. Just over 40% of children did not accumulate 60 minutes per day of MVPA, the physical activity guideline for children at the time [6,39]. However, a large proportion (~90%) of these children’s mothers perceived their child to be active. These findings suggest that mothers of inactive children do not, in general, perceive their child as such. If parents are unaware that their children are inactive, they may be less inclined to encourage physical activity or participate in interventions to promote it. When compared with mothers who accurately perceive their child as active, mothers were more likely to overestimate physical activity levels if their child attended nursery full time and if they perceived that their child on occasion lacked the necessary skills to participate in activities. In addition, mothers of children without older siblings were more likely to overestimate their child’s physical activity level.

The proportion of mothers in this study who overestimated their four-year-old’s activity level (~90%) was slightly greater than that previously reported in parents of 9 and 10 year-old children (~80%) [25,26]. Mothers here also overestimated their child’s activity levels to a greater extent than adults have for their own activity [21,22,49]. It is possible that mothers overestimated activity levels here due to social desirability bias or because they were asked if they thought that their child was active, not whether their child engaged in 60 minutes of activity each day. This is a limitation of this study, but that two previous studies found similar levels of overestimation in parents of older children regardless of whether an average [25] or daily measure [26] of activity and parental perception was used, provides confidence in the validity of the findings presented here. Further, given that adults have been found to overestimate their own activity levels [21,22,49], and that only a moderate correlation exists between parental proxy reports and objectively measured physical activity [50], it is conceivable that many parents may not be aware of their child’s low level of physical activity.

Nursery attendance was independently associated with maternal overestimation: mothers were more likely to overestimate activity levels if their child attended nursery or preschool full time. Children now spend increasing amounts of time in preschool or nursery education, with studies showing that children in these settings are sedentary for a large proportion of the time [12]. It is plausible that mothers may hold unrealistic expectations or be over-optimistic about their child’s activity levels when they spend prolonged periods away from their child. An increased understanding of how physical activity levels differ according to whether a child is in a child-care setting, and how time spent at preschool is associated with physical activity levels would help to understand this observation and define intervention targets.

Mothers with one or more older child at home were less likely to overestimate their four-year old’s activity level. It is possible that having siblings older than the sample children provide mothers with a reference point on which to base their assessment of their four-year-old’s activity. However, it is difficult to disentangle this from the possibility that children with older siblings may be more active, as shown previously in older children [51,52]. The association found between maternal perception that their child on occasion lacks the necessary skills to be physically active and overestimation is also interesting. Although this may be a chance finding, adults who overestimate their activity levels have previously been shown to have similar scores on psychological factors as those who are realistic [21]. Therefore mothers who acknowledge that their child on occasion lacks the skills required to be physically active may still believe that this does not affect their child’s overall level of activity, leading to its overestimation.

We did not find an association between child’s BMI z-score and maternal overestimation of activity. The absence of an association between BMI and overestimation in these analyses is in line with previous research, as we only compared inactive and active children of mothers who considered their child to be active. Previous awareness literature has predominantly compared over-estimators with those accurately reporting their own, or their child’s, inactivity. These studies consistently show that those with a (child with a) more favourable body composition tend to overestimate activity levels [21,22,25]. This association is hypothesized to occur because over-estimators assume a favourable body composition is indicative of sufficient physical activity. As there is no clear evidence of the association between physical activity and BMI (z-score) in preschool children [23], it is plausible that weight may be less salient to mothers when considering their child’s activity levels at this age. The percentage of overweight (8.6%) and obese (3.2%) children in this sample was lower than the English average (13% and 9.6% respectively) [53], but as mothers were not asked their perception of their child’s BMI, it is not possible to determine whether this was the case here.

A clear understanding of the influence parents have on their children's physical activity behaviour is required for the development of effective family-based interventions to promote physical activity in preschool children [54]. Given the majority of mothers of inactive children were unaware of their child’s inactivity, they may be less likely to encourage their child to engage in activity. As younger children do not possess the ability to adequately monitor their own behaviour [29], they must rely instead on parental proxy monitoring. It is interesting that regardless of activity level, mothers who deemed their child to be active held differing perceptions about a child’s general and activity behaviour from mothers who did not. Although far fewer mothers fell into the latter category, data presented here suggest that factors such as child temperament and mothers’ own perceptions about activity will also influence awareness. Improving this activity awareness may therefore be facilitated by use of monitors such as pedometers, whilst at the same time encouraging greater physical activity in children [55]. Use of pedometers in preschool children is feasible, with moderate correlations reported with accelerometry and direct observation to measure activity [50]. However, research is required to determine how different levels of parental awareness affect intervention success and whether monitoring evokes a positive change in preschool children’s behaviour. Nonetheless, maternal or parental awareness may be important to consider when trying to increase levels of physical activity in younger children.

### Study limitations and strengths

The authors are unaware of any previous studies that have looked at factors associated with maternal awareness of physical activity levels in preschool children. Preschool children are a relatively under-studied age group, and a wide range of potential correlates of overestimation of physical activity were explored based on their plausible influence. Objective accelerometry data was used to measure activity levels in the children and then combined with data from a validated questionnaire to derive awareness categories. Accelerometry is a valid measure in this age group [56] and captures activity levels more accurately than when assessed by proxy in questionnaires or using pedometers [50].

As this study was cross-sectional, it is not possible to determine the direction of causality for the associations found. Also, due to storage capacity at time of measurement, an epoch of 60 seconds was used in this study. Previous evidence indicates that this may have contributed to an underestimation of activity [57]. Although it is difficult to assess the impact of this on the conclusions drawn here, it will result in a high specificity for capturing MVPA, which is likely to better reflect mothers’ perceptions of their children’s physical activity. For this reason, we also used the previous activity guideline of 60 minutes of MVPA each day, the standard at the time of measurement [6], to classify children as in/active. This increased specificity between maternal-reported activity levels, likely to be answered with reference to this guideline, and objectively measured physical activity. Although this makes comparison with current guidelines more challenging, it contributes to the wider public health debate as to the relevance of activity intensity in young children. As MVPA has been shown to provide favourable health outcomes in young children [15,16], it remains important for parents to be aware of their child’s MVPA levels. No significant differences were found between those included and excluded, but attrition from the original sample may prevent it being truly representative of the original cohort. Further, fewer children in this sample were overweight or obese compared to the national average and participants were predominately white British in keeping with the Southampton region (~82%) [58]. This suggests that the sample is not representative of the British population of preschool children as a whole, reducing the generalizability of the findings.

## Conclusions

A large proportion of mothers of inactive preschool children inaccurately perceive their child to be active. Promoting awareness of activity levels in mothers of children who spend large amounts of time at nursery school, who perceive their child on occasion lacks physical activity skills, or who do not have older siblings may be particularly important. These children may require more parental input to promote behaviour change and increase their activity levels. However, further research is also needed to investigate the effect of changes in awareness on behaviour change.

## Abbreviations

PA: Physical activity; MVPA: Moderate and vigorous physical activity; BMI: Body mass index.

## Competing interests

The authors declare that they have no competing interests.

## Authors’ contributions

NH, HI, KG and CC were involved in the setting up and running the SWS Study; all authors were involved in the acquisition of data for this analysis; KH, AM, and EvS developed the research question and analysis design; all other authors provided input on the analysis design. KH conducted the analyses and drafted the manuscript; all authors were involved in interpreting the data and revising the manuscript, and have approved the final version for publication.

## Pre-publication history

The pre-publication history for this paper can be accessed here:

http://www.biomedcentral.com/1471-2458/13/924/prepub
